# Reduced empathic concern leads to utilitarian moral judgments in trait alexithymia

**DOI:** 10.3389/fpsyg.2014.00501

**Published:** 2014-05-26

**Authors:** Indrajeet Patil, Giorgia Silani

**Affiliations:** Cognitive Neuroscience Sector, Scuola Internazionale Superiore di Studi AvanzatiTrieste, Italy

**Keywords:** moral judgment, alexithymia, utilitarianism, moral dilemma, empathy, empathic concern

## Abstract

Recent research with moral dilemmas supports dual-process model of moral decision making. This model posits two different paths via which people can endorse utilitarian solution that requires personally harming someone in order to achieve the greater good (e.g., killing one to save five people): (i) weakened emotional aversion to the prospect of harming someone due to reduced empathic concern for the victim; (ii) enhanced cognition which supports cost-benefit analysis and countervails the prepotent emotional aversion to harm. Direct prediction of this model would be that personality traits associated with reduced empathy would show higher propensity to endorse utilitarian solutions. As per this prediction, we found that trait alexithymia, which is well-known to have deficits in empathy, was indeed associated with increased utilitarian tendencies on emotionally aversive personal moral dilemmas and this was due to reduced empathic concern for the victim. Results underscore the importance of empathy for moral judgments in harm/care domain of morality.

“Interesting, yes, emotions. The grit on the lens, the fly in the ointment.”

– Sherlock Holmes (*Sherlock*)

## Introduction

### Moral dilemmas and dual-process model

The aversion to harming others is an integral part of the foundations of human moral sense and it presents itself in the form of deeply ingrained moral intuitions (Haidt and Joseph, 2004). Since creating laboratory situations to investigate harm aversion raises ethical issues, research has primarily relied on studying hypothetical cases like moral dilemmas where participants need to give judgments about whether they would be willing to harm one person so that many others would go unhurt. Moral dilemmas are ideal for the purpose of studying harm aversion because the type of harm and the means of carrying out this harm can be systematically varied in these situations to see how variations in these factors affects moral judgments (Christensen and Gomila, 2012; Trémolière and De Neys, 2013). For example, the trolley dilemma (Thomson, 1985) asks participants to judge if it is appropriate for them to switch a trolley hurtling down on a track toward five people onto an alternate track where there is just one person, while the footbridge dilemma (Thomson, 1985) asks participants to judge if it is appropriate for them to push a large person standing near them off of a footbridge, so that this individual would fall down to his/her death and collide with the trolley, stopping it from running over five people down the track. People who endorse switching the trolley or pushing the person are said to have taken utilitarian decision because utilitarianism (Mill, 1863/1998) argues that it is morally acceptable to cause harm to few if this is going to prevent a greater number of people from getting hurt. On the other hand, people who refuse to accept switching the trolley or pushing the person are said to have taken deontological decision because deontology (Kant, 1785/2005) argues that individuals have inviolable right and duties which can't be infringed upon even if doing so would maximize welfare of more number of individuals. These two dilemmas respectively are quintessential examples of two classes of moral dilemmas: impersonal and personal moral dilemmas (Greene et al., 2001, 2004, 2009). They differ in their structural descriptions and mental representations (Mikhail, 2007). In personal moral dilemmas, the victim needs to be harmed using personal force (i.e., by executing a motor act) and this harm is intentional rather than a side-effect (Cushman et al., 2006; Greene et al., 2009). Thus, footbridge dilemma is an example of a personal moral dilemma where the victim is pushed via muscular force and its death is *needed* for the utilitarian outcome to materialize and is, thus, not a side-effect. On the other hand, trolley dilemma is an example of an impersonal moral dilemma where the harm is caused in an impersonal way by pushing the switch and death of the victim is a side-effect of switching the trolley on the alternate track and not a means by which the lives of five are being saved. Although the net outcome of choosing to act in both types of dilemmas is the same (four net lives saved), most people endorse utilitarian solutions on impersonal dilemmas but refuse to do so on personal dilemmas (Greene et al., 2001, 2004, 2008, 2009; Cushman et al., 2006; Hauser et al., 2007; Mikhail, 2007; Moore et al., 2008; Gleichgerrcht and Young, 2013).

The most influential model to account for these findings has been proposed by Joshua Greene and colleagues (Greene et al., 2001, 2004, 2008, 2009; Greene, 2007, in press; Shenhav and Greene, 2014), called dual-process model, which posits two set of computational processes that support these two competing moral ideologies: (1) deliberative reasoning processes that engage in cost-benefit analysis by a conscious weighing of different social norms and situational factors and support utilitarian solution; (2) automatic, affect-laden intuitions that surface as a reflex to aversive nature of the proposed harm and subserve deontological intuitions. Thus, according to Greene's model, when people think about moral dilemmas and face the question as to whether one person should be sacrificed so that many more would be better off, they experience an aversive, negative emotional reaction to the prospect of actively harming someone physically. This aversion can partially stem from the bad outcome due empathic concern for the victim's pain which causes personal distress in the observer/actor (Pizarro, 2000; Miller and Cushman, 2013) and partially from performing the bad action itself due to history of aversive conditioning associated with the sensorimotor and perceptual properties of the action (Cushman et al., 2012; Miller et al., 2014). If this prepotent, negative affect stemming from harm aversion is strong enough and is not countervailed by deliberative reasoning processes, deontological inclinations would prevail and people would judge it wrong to sacrifice one person to save five, as in personal moral dilemma. On the other hand, if there is not a strong emotional reaction to the prospect of harming someone, then the controlled cognition would dominate the decision making process and would lead to endorsement of utilitarian solution, as in impersonal moral dilemma. It is important to note that the two processes proposed by dual-process model are independent processes that contribute to the final outcome and are not inversely proportional to each other (Conway and Gawronski, 2013). Thus, one can find it morally acceptable to personally harm someone in order to achieve the greater good either because they are better at cognitive deliberation (e.g., abstract reasoning, problem solving, etc.) and find it pragmatically more acceptable after cost-benefit analysis *or* because they have a blunted sense of harm aversion due to reduced empathic concern for the victim. There is plenty of evidence to corroborate the claim that people take both of these routes when they make utilitarian decisions on moral dilemmas.

### Two paths to utilitarian moral judgments

People who report to have higher need for cognition, i.e., people who say they enjoy engaging in deliberate reasoning, tend to be more utilitarian (Bartels, 2008). Also, people with higher working memory capacity, which provides the necessary cognitive resources for cognitive deliberation, prefer utilitarian solutions for moral dilemmas (Moore et al., 2008). People who perform better on cognitive reflection task, which assesses individual's propensity to distrust intuitions in favor of reflective and deliberative processes, also prefer utilitarian solutions (Paxton et al., 2012, 2013; Baron, 2013; but see Royzman et al., in press). Disrupting cognitive processing by imposing cognitive load or by using noninvasive brain stimulation technique makes participants either slow down while endorsing utilitarian solutions (Greene et al., 2008) or makes it less likely that they will endorse utilitarian solutions (Trémolière et al., 2012; Conway and Gawronski, 2013; Jeurissen et al., 2014; but see Tassy et al., 2012). Easing up cognitive demands by using efficient kill-save ratios makes people more utilitarian (Trémolière and Bonnefon, 2014). Cognitively exhausting participants using sleep deprivation also increases response latencies while providing utilitarian moral judgments (Killgore et al., 2007; Tempesta et al., 2011). Forcing participants to respond as quickly as possible without giving sufficient time for deliberative reflection to weigh in makes participants more deontologically inclined (Suter and Hertwig, 2011; Cummins and Cummins, 2012). Stress is well-known to inhibit cognitive control and affect working memory capacity, which are the very cognitive resources needed to make utilitarian moral judgments. Accordingly, stressed participants are less likely to endorse utilitarian solutions than unstressed participants (Starcke et al., 2012; Youssef et al., 2012). Thus, existing studies support the presence of reflective reasoning path that leads to utilitarian moral judgments.

On the other hand, there is also evidence corroborating the claim that blunted negative affect due to reduced empathy for the victim in the dilemma can lead to the utilitarian solution. Meta-analysis of brain imaging studies shows that moral cognition recruits subset of the brain areas involved in empathy (Bzdok et al., 2012; Sevinc and Spreng, 2014) and damage to these areas results in aberrant empathic skills and moral judgments. Patients with damage to ventromedial prefrontal cortex (vmPFC, a brain region essential for proper emotional processing), frontal traumatic brain injury patients, and patients suffering from behavioral variant of frontotemporal dementia (bvFTD, which also includes deterioration of frontal lobes) are known to possess uncallous emotionality, shallow social affect, and tend to lack empathy. All of these populations are more likely to endorse utilitarian solutions on high-conflict, personal moral dilemmas (Mendez et al., 2005; Ciaramelli et al., 2007; Koenigs et al., 2007; Mendez and Shapira, 2009; Moretto et al., 2010; Gleichgerrcht et al., 2011; Thomas et al., 2011; Martins et al., 2012; Chiong et al., 2013; Taber-Thomas et al., 2014) than brain-damaged and neurotypical control populations. This is probably because they find the prospect of personally harming someone less emotionally aversive due to reduced empathic response, as shown by reduced skin conductance arousal in vmPFC patients when they face personal moral dilemmas (Moretto et al., 2010) and reduced emotional empathy on self-report measures in bvFTD patients (Gleichgerrcht et al., 2011). Core aspects of psychopathy are also associated with lack of empathy and shallow affect and both incarcerated, clinical psychopaths (Koenigs et al., 2012) and nonincarcerated individuals with psychopathic tendencies show predilection for utilitarian solutions on personal moral dilemmas (Glenn et al., 2010; Bartels and Pizarro, 2011; Langdon and Delmas, 2012; Gao and Tang, 2013; Seara-Cardoso et al., 2013; Tassy et al., 2013; Djeriouat and Trémolière, 2014). One behavioral study shows that justifications given by psychopathic personalities for utilitarian moral judgments involve less inclusion of empathic terms (McIlwain et al., 2012), while brain imaging studies show that these increased utilitarian dispositions in psychopathy are due to reduced activity in subgenual anterior cingulated cortex (Wiech et al., 2013), which is implicated in empathic concern for others. People who score high on trait emotional empathy also show reduced tendency to endorse personal harms and resort to deontological responses (Choe and Min, 2011), while self-reported or peer-reported low scores on dispositional empathic concern (which measures individual's tendency to experience feelings of warmth, compassion, and concern for other people), predict higher proportion of utilitarian moral judgments (McIlwain et al., 2012; Côte et al., 2013; Gleichgerrcht and Young, 2013; Jack et al., 2014; Miller et al., 2014) and higher unpleasantness ratings for both impersonal and personal moral dilemmas (Sarlo et al., 2014). Also, enhancing the empathic concern for the would-be victims by showing their photographs (Conway and Gawronski, 2013), highlighting their humanness (Majdandžić et al., 2012), emphasizing their competency (Cikara et al., 2010), or drawing attention to age of the sacrificial target (Kawai et al., 2014) makes people less inclined toward utilitarian decisions. Making people emotionally more averse to perceived harmful acts by pharmacologically enhancing serotonin levels in the brain lessens frequency of decisions that endorse utilitarian ends and, more interestingly, this effect is especially stronger for people scoring higher on empathy (Crockett et al., 2010; also see Terbeck et al., 2013). On the other hand, higher level of testosterone (either baseline level or after external administration) has been associated with impairments in empathic behavior and reduced negative social emotions and is associated with utilitarian moral judgments for personal moral dilemmas (Carney and Mason, 2010; Montoya et al., 2013). This is probably because high-testosterone individuals are less sensitive to the emotionally salient nature of physical harm (Carney and Mason, 2010). Given this overwhelming evidence for the role of reduced empathy in making utilitarian moral judgments, it is of value to study populations which have known empathy deficits to see if they show increased predisposition toward utilitarianism. One such population is alexithymia to which we turn next.

### Alexithymia and empathy deficits

Alexithymia, or “no words for feeling,” is a dimensional personality construct that is characterized by reduced capacity to experience emotions, absence of tendency to reflect on one's own emotions, difficulty in identifying feelings and bodily sensations associated with emotional arousal, and describing these feelings to others (Nemiah et al., 1976), e.g., individuals with alexithymia might be aware that they are experiencing an emotion, but would be unable to pinpoint if the emotion is anger, sadness, or disgust. Given the critical role of emotion in effective social behavior like perception and evaluation of socio-emotional stimuli and regulation and modulation of social behavior according to such evaluations, alexithymic population performs poorly on a number of social cognition tasks, e.g., empathy, emotion recognition, emotional interoception, etc. (Wingbermühle et al., 2012; Bird and Cook, 2013). Of interest to the current study are problems associated with empathy in alexithymic personalities.

Empathy is composed of two separate and equally important components: (i) cognitive empathy involves understanding others' emotional states by forming abstract mental representations of these states while maintaining self-other distinction; (ii) affective empathy involves experiencing these emotional states (de Vignemont and Singer, 2006). In other words, affective empathy entails that we share the isomorphic affective state of the target (“I suffer, because you suffer”), while cognitive empathy involves merely representing these affective states without necessarily experiencing them (“From my observation of your behavior, I infer that you are suffering”). Recent work in social neuroscience supports the shared network model of empathy which posits that the same brain regions that are involved in mapping body's physiological states that inform of us of our subjective feelings states are also involved when we try to predict the feeling states of others (Decety and Sommerville, 2003; Singer and Lamm, 2009; for meta-analytic evidence, see Lamm et al., 2011). In other words, when people try to understand emotional states of others and experience these states vicariously, they are guided by their own internally generated affective states (Hooker et al., 2008). But this very ability to identify and describe feelings and interocepting on one's emotions is compromised in alexithymia (e.g., Silani et al., 2008). Because awareness of emotional states in the self is a prerequisite to recognizing such states in others, reduced capacity in alexithymia to recognize and attend to one's own affective state is expected to lead to impairment in empathizing with others.

Indeed, high level of alexithymia is associated with reduced activity in the empathy circuits when they empathize with others who are experiencing pain (Moriguchi et al., 2007; Bird et al., 2010). They also report to feel less distress at others' suffering and are less motivated to act altruistically to relieve another's distress (FeldmanHall et al., 2012). Various self-report measures of empathy, e.g., IRI (interpersonal reactivity index: Davis, 1980, 1983), show that alexithymic personalities report to have less empathic concern for others and reduced tendency for perspective-taking in both community and psychiatric/clinical populations (for a review, see Bird and Cook, 2013; also see Guttman and Laporte, 2002; Grynberg et al., 2010). They also show reduced empathic response to emotional facial expressions (Lockwood et al., 2013). Thus, there is overwhelming evidence that trait alexithymia is characterized by poor ability to understand what others feel (cognitive) and experience or share others' emotional states (affective).

### Current study

To summarize the discussion so far, dual-process model of decision-making predicts that deflated negative affect due to reduced empathic concern for the victim distress when someone thinks about personally harming the victim can lead to utilitarian moral judgments and alexithymia is associated with reduction in this very aspect of empathy. In this study, we extend this work by exploring the utilitarian tendencies associated with trait alexithymia and role of empathy in this association and make three key predictions:

1. Higher level of trait alexithymia will predict reduced empathic concern and increased acceptance of utilitarian choice on personal moral dilemma.
2. Reduced empathic concern will predict higher acceptability of the utilitarian option for personal moral dilemma.
3. Empathic concern scale of IRI will mediate the relation between trait alexithymia and acceptability of utilitarian choice on personal moral dilemma.

We would not expect to see any increased tendency in trait alexithymia to make utilitarian choices on impersonal moral dilemma because the nature of harm in this dilemma is emotionally less salient and does not invoke the prepotent empathic response with the victim as the personal moral dilemma where the agent (participant) needs to agree to harm someone personally. Also, we do not expect alexithymic impairment in cognitive empathy (as assessed by perspective-taking subscale of IRI) to play role in the alexithymia-utilitarian association because a number of previous studies show that perspective-taking does not predict utilitarian moral judgments (McIlwain et al., 2012; Côte et al., 2013; Gleichgerrcht and Young, 2013; Jack et al., 2014; Miller et al., 2014; Sarlo et al., 2014).

## Materials and methods

### Participants

Three hundred and thirty one (215 women) Italian-speaking participants between the ages of 18 and 60 (*M* = 24.06, *SD* = 5.50) voluntarily logged on to fill a web survey. The survey webpage was promoted through discussion on online forums, social network, and word of mouth. Exclusion criteria for participation included Italian as a secondary language, presence of a diagnosed psychiatric illness and/or history of psychiatric treatment, history of significant neurological illness or brain injury.

### Measures and procedure

All participants gave an informed consent before starting the survey. They then progressed through a series of self-report measures that assessed variables of interest and answered two moral dilemmas (one impersonal and one personal). The order in which participants completed the various questionnaires was randomized across participants. But since we were not interested in studying transfer effects between dilemmas (e.g., Wiegmann and Waldmann, 2014), the order of moral dilemmas was fixed such that personal moral dilemma always preceded the impersonal moral dilemma. There was no time limit to answer any of the questionnaires or dilemmas. All the questionnaires and dilemmas provided were in Italian and the translated documents are available upon request to the corresponding author.

#### Moral judgment

Participants were presented with a pair of moral dilemmas, each of which required participants to choose whether to harm one person to save five people (see Supplementary Text S1 for exact wording of dilemmas). Personal moral dilemma was Footbridge dilemma which featured emotionally aversive harm (pushing the person to his/her death). Impersonal moral dilemma was Standard Fumes (Greene et al., 2004) and featured less emotionally salient harm (hitting a switch which would divert toxic fumes from room with five patients to room with just one patient). Both dilemmas were framed in first-person and asked the question “How morally appropriate is it for you to [nature of action] in order to [outcome of action]?” Participants could register their answer using a 7-point Likert scale (1: *not at all*, 7: *very much*). Higher appropriateness scores denoted more utilitarian inclination.

#### Alexithymia

To assess trait alexithymia, we used validated Italian version of Toronto Alexithymia Scale-20 (TAS-20) questionnaire (Bagby et al., 1994; Italian version: Bressi et al., 1996), which has been argued to be the best current measure overall for assessing alexithymia due to its sound reliability, validity, and broad generalizability (Timoney and Holder, 2013). The TAS-20 is a 20-item scale that consists of three subscales: Difficulty Describing Feelings (DDF, 5 items, e.g., “It is difficult for me to find the right words for my feelings”), Difficulty Identifying Feelings (DIF, 7 items, e.g., “When I am upset, I don't know if I am sad, frightened, or angry”), and Externally-Oriented Thinking (EOT, 8 items, e.g., “I prefer to analyze problems rather than just describe them”). Items were rated using a 5-point Likert scale (1: *strongly disagree*, 5: *strongly agree*). Higher total scores indicate higher levels of alexithymia.

#### Empathy

Participants completed validated Italian version of Interpersonal Reactivity Inventory (IRI) (Davis, 1980, 1983; Italian version: Albiero et al., 2006), a 28-item self-report questionnaire with four 7-item subscales, assessing specific aspects of dispositional empathy. Participants reported agreement with statements on a 5-point Likert scale (0: *never true for me*, 4: *always true for me*). The four subscales consisted of: (1) fantasy scale (F), which measures the propensity to identify with fictional characters (e.g., “I really get involved with the feelings of the characters in a novel.”); (2) perspective taking (PT) scale, which measures the tendency to take the psychological point of view of others (e.g., “I try to understand my friends better by imagining how things look from their perspective.”); (3) empathic concern (EC) scale, which measures the *other-oriented* tendency to experience feelings of warmth, compassion, and concern for other people (e.g., “When I see someone being taken advantage of, I feel kind of protective toward them.”); (4) personal distress (PD) scale, which measures the *self-oriented* tendency to feel personal unease and discomfort in reaction to the emotions of others (e.g., “When I see someone who badly needs help in an emergency, I go to pieces.”). Thus, IRI defines empathy to be a multidimensional construct consisting of cognitive ability to take others' perspective (PT) and understand their subjective reality (FS) and affective ability to put oneself in others' emotional shoes to experience concern for their wellbeing (EC) and be affected by their experiences (PD).

### Data analysis

Statistical analysis was conducted using SPSS 22.0 software. Given recent criticism of dichotomous logic of null-hypothesis testing and poor reproducibility of *p*-values in psychological research (Ioannidis, 2005), we include recommended confidence intervals for estimator values and effect sizes (Cumming, 2014) generated using resampling and bootstrapping methods (Kirby and Gerlanc, 2013). Unless otherwise stated, all 95% bias corrected and accelerated confidence intervals were generated using 10,000 bootstrap samples. If present, asymmetry in 95% bias corrected and accelerated confidence intervals reflects asymmetry of the underlying sampling distribution of point estimates. We also include traditional *p*-values, all of which are exact rather than based on asymptotic approximation and computed from two-tailed statistical tests.

Since the dependent variables of interest (scores on IRI subscales, and acceptability ratings for moral dilemmas) did not follow normal distribution (Shapiro-Wilk test: *p* < 0.01) and were ordinal variables, all tests employed were nonparametric. We used ordered logistic regression models when regression was of interest to us instead of linear regression. Test of parallel lines showed that none of the regression models violated the proportional odds assumption (*p* > 0.05). We report *unstandardized* logit coefficients (*B*) from which odds ratios can be computed using exponential function as *e^B^*. We don't report and compute odds ratios from *standardized* logit coefficients because there is no widely agreed upon definition of it, thereby preventing straightforward interpretation (Hosmer and Lemeshow, 2004). Odds ratio greater than 1 or less that 1 denote that increase in value of predictor variable is associated with increased likelihood for *higher* or *lower* value of criterion variable, respectively. Also, for inter- and intra-group comparisons, Mann-Whitney U test and Wilcoxon Signed Rank test were used and effect size (*r*) for these tests was computed as *r = Z/√n*, where *Z* is the standardized statistic and *n* is the sample size (Fritz et al., 2012). Additionally, we report Hodges–Lehmann (HL) estimator values for the median difference between groups being compared. Correlation analysis was done using Spearman rank correlations and, when necessary, partial Spearman rank correlations were computed using SPSS syntax (see: http://www-01.ibm.com/support/docview.wss?uid=swg21474822).

For mediation analysis, we did not use Sobel's test because: (a) it has poor statistical power and is not recommended for small sample sizes *n* < 1000, MacKinnon et al. (2002) which was the case for our study (*n* = 331); (b) it evaluates samples on the assumption that indirect effects follow normal distribution, which is hardly true in practice. We instead used nonparametric, Preacher-Hayes bootstrapping method to estimate indirect effects in mediation analysis because statistical power-wise it is more robust with small sample sizes (*n* < 25) and it does not assume normal distribution for indirect effects (Preacher and Hayes, 2004, 2008a).

Because sufficient power is required to claim meaningful null effects (i.e., TAS, subscales of IRI, and ratings on moral dilemmas are not correlated with each other), we conducted a sensitivity analysis using G^*^Power 3.1 (Faul et al., 2007) to determine how small of an effect we could detect in each correlation analysis (exact two-tailed tests, see Supplementary Tables S1,2). With a sample size of 331, a Type II error probability of α = 0.05, and a statistical power (Type I error probability) of β = 0.80, we could detect an effect size > 0.153 (i.e., a small effect; Cohen, 1992). We could not perform a similar analysis for ordinal logistic regression analysis due to unavailability of this option in G^*^Power.

## Results

### Descriptive statistics (table 1)

The subscales of TAS-20 and IRI showed good internal reliability (αs > 0.60). In our sample, mean alexithymia score was 44.58 (95% CI [43.42, 45.71]) with a minimum-maximum spread of 20–71. It has been reported (Loas et al., 2001) that alexithymia in normal population follows distribution with mean of 45 (*SD* = 8). Thus, our sample was within normative range (one-sample z-test: *Z* = −0.955, *p* = 0.339). When alexithymia is treated as a categorical construct (vis-à-vis dimensional personality trait), individuals with TAS scores equal to or greater than 61 are considered to be alexithymic (*n* = 30), between 52 and 60 are considered to be possibly alexithymic (*n* = 60), and equal to or less than 51 are considered to be nonalexithymic (*n* = 241) (Bagby et al., 1994). In the following discussion, we will provide results only from the dimensional perspective. For the same analysis with categorical construct, see Supplementary Text S2.

**Table 1:** **Means with 95% confidence interval values, medians, minimum-maximum spread, gender differences, and Cronbach alphas for alexithymia, empathy scores, and moral dilemma judgments**.

**Items**	**Cronbach alpha**	**Mean [95% CI]**	**Median**	**Min, Max**	**Gender effects (*Z*)**
DDF	0.665	12.51 [12.06, 12.95]	12	5, 24	−0.824
DIF	0.778	16.73 [16.15, 17.31]	16	7, 32	2.267^*^
EOT	0.629	15.34 [14.86, 15.86]	15	8, 28	−1.760
TAS-20	0.818	44.58 [43.42, 45.71]	44	20, 71	0.399
F	0.794	17.67 [17.21, 18.11]	18	5, 28	3.725^***^
PT	0.835	18.05 [17.57, 18.53]	18	3, 28	1.070
EC	0.781	18.69 [18.26, 19.10]	19	7, 28	4.295^***^
PD	0.802	11.35 [10.85, 11.87]	11	0, 26	4.175^***^
IRI-total	0.841	65.77 [64.71, 66.88]	66	31, 95	5.509^***^
Impersonal	–	4.56 [4.34, 4.79]	5	1, 7	−2.692^**^
Personal	–	2.51 [2.31, 2.74]	2	1, 7	−1.432

*SD, standard deviation; DDF, difficulty describing feelings; DIF, difficulty identifying feelings; EOT, externally-oriented thinking; TAS, Toronto Alexithymia Scale; F, fantasy; PT, perspective taking; PD, personal distress; EC, empathic concern; IRI, Interpersonal Reactivity Index. -, not applicable. Z, standardized statistic from Mann-Whitney U test. Positive value of Z signifies that women scored higher on this variable than men*.

* p < 0.05;

** p < 0.01;

*** *p < 0.001*.

There was strong effect of gender for IRI scores; women scored higher on fantasizing, empathic concern, and personal distress (*r*s > 0.180, HL estimators = 2.000 [1.000, 3.000]). But both genders reported of being equally capable of perspective taking (*p* > 0.3).

As expected, the acceptability judgment for impersonal moral dilemma was higher than for personal moral dilemma (Wilcoxon Signed Ranks test: *Z* = 12.793, *p* < 0.001, *r* = 0.703, HL estimator = 2.000 [2.000, 2.500]). Thus, people found it more acceptable to endorse utilitarian option in impersonal than personal moral dilemma. Additionally, women gave lower appropriateness rating than men for impersonal moral dilemma (*r* = −0.148, HL estimator = 1.000 [0.000, 1.000]). As expected, there was more variation for moral judgments about personal (coefficient of variation = 73.4%) as compared to impersonal (coefficient of variation = 41.2%) moral dilemma nonparametric Levene's test: *F*_(1,660)_ = 127.8, *p* < 0.001.

### Regression analysis (tables 2, 3)

Ordinal regression with TAS as the predictor variable (see Table 2) showed that increase in trait alexithymia was associated with higher likelihood of reporting *higher* personal distress with odds ratio of 1.051 (95% CI [1.032, 1.069]). Higher scores on trait alexithymia also predicted an increase in odds of reporting *lower* perspective taking, with an odds ratio of 0.982 (95% CI [0.964, 1.002]), and empathic concern, with an odds ratio of 0.979 (95% CI [0.962, 0.996]). Also, a unit increase in trait alexithymia increased odds of higher appropriateness rating for personal moral dilemma (see Supplementary Figure S2) with an odds ratio of 1.025 (95% CI [1.006, 1.048]), but there was only marginally significant association between alexithymia and ratings for impersonal moral dilemma (odds ratio = 1.0171 [0.9960, 1.0356], *p* = 0.058).

**Table 2:** **Alexithymia (TAS) scores predicting judgments on moral dilemmas and empathy IRI subscales**.

**Predictor variable**	**Criterion variable**	Logit coefficient [95% CI]^*^	**Wald's chi-square**	***p*-value**
TAS-20	F	0.013 [−0.004, 0.036]	2.125	0.145
	PT	−0.018 [−0.037, 0.002]	4.063	0.044
	EC	−0.021 [−0.039, −0.004]	5.675	0.017
	PD	0.050 [0.032, 0.067]	29.890	<0.001
	Impersonal	0.017 [−0.004, 0.035]	3.602	0.058
	Personal	0.025 [0.006, 0.047]	7.434	0.006

*TAS, Toronto Alexithymia Scale; F, fantasy; PT, perspective taking; PD, personal distress; EC, empathic concern; CI, confidence interval. See Supplementary Table S3 for the same analysis with age and gender as additional predictor variables*.

* *95% bias corrected and accelerated confidence intervals for logit coefficients were generated using 10,000 bootstrap samples. Positive or negative value of logit coefficient denote that increase in value of predictor variable is associated with increased odds for higher or lower value of criterion variable, respectively*.

Ordinal regressions with subscales of IRI (see Table 3) showed that only scores on empathic concern showed any association with ratings on moral dilemmas. Increase in self-reported empathic concern for others was associated with increase in odds of finding utilitarian option on personal moral dilemma to be *less* appropriate with an odds ratio of 0.935 (95% CI [0.885, 0.983]) for personal moral dilemma. There wasn't any significant association between empathic concern and impersonal moral dilemma (*p* = 0.341).

**Table 3:** **IRI subscale scores predicting judgments on moral dilemmas**.

**Predictor variable**	**Criterion variable**	Logit coefficient [95% CI]^*^	**Wald's chi-square**	***p*-value**
F	Impersonal	−0.009 [−0.050, 0.034]	0.190	0.663
	Personal	0.020 [−0.028, 0.068]	0.744	0.388
PT	Impersonal	0.002 [−0.045, 0.045]	0.009	0.925
	Personal	0.012 [−0.028, 0.055]	0.283	0.595
EC	Impersonal	−0.023 [−0.076, 0.027]	0.907	0.341
	Personal	−0.067 [−0.122, −0.017]	6.737	0.009
PD	Impersonal	0.000 [−0.043, 0.042]	<0.000	1.000
	Personal	0.010 [−0.036, 0.057]	0.228	0.633

*F, fantasy; PT, perspective taking; PD, personal distress; EC, empathic concern; CI, confidence interval. See Supplementary Table S4 for the same analysis with age and gender as additional predictor variables*.

* *95% bias corrected and accelerated confidence intervals for logit coefficients were generated using 10,000 bootstrap samples. Positive or negative value of logit coefficient denote that increase in value of predictor variable is associated with increased odds for higher or lower value of criterion variable, respectively*.

All of the above-mentioned results held even after controlling for effects of age and gender by including them in predictor variables (see Supplementary Tables S3–4). Correlation analysis further upheld the results from regression analysis and painted the same picture further corroborating our two predictions (see Supplementary Tables S1,2).

### Mediation analysis (figure 1)

**Figure 1:**
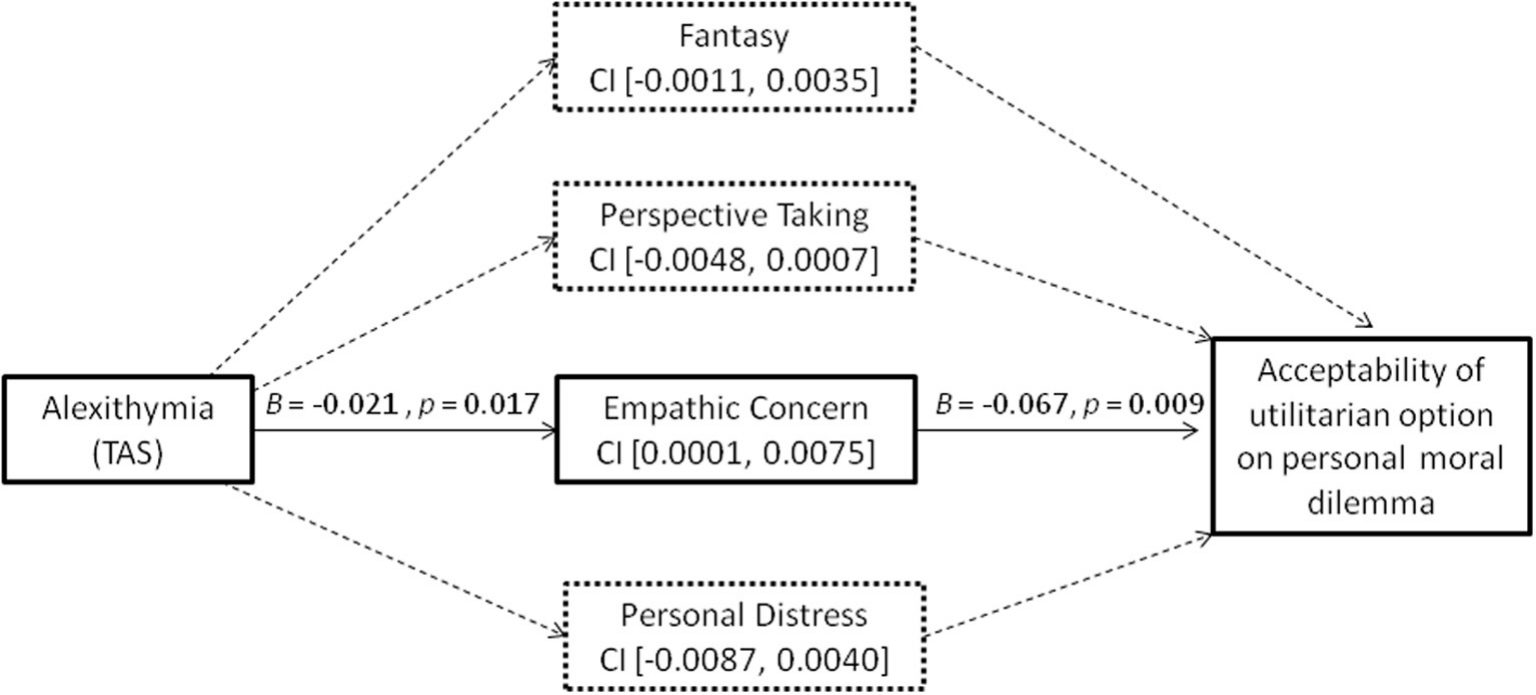
**Mediation analysis results**. Negative logit coefficient from ordinal regression denotes reduced empathic concern and increased acceptability of utilitarian option on personal moral dilemma. Bias-corrected and accelerated 95% CIs from 20,000 bootstrap samples are reported for specific indirect effects. The increased utilitarian tendency on personal dilemma in trait alexithymia was due to reduced empathic concern. Continuous lines denote significant mediation path, while dashed lines denote nonsignificant mediation path.

Mediation analyses were used to ascertain the degree to which increased utilitarian tendency in trait alexithymia on personal moral dilemma was mediated through indirect effects stemming from decreased levels of empathic concern in this trait. Bootstrap estimation of 95% bias-corrected and accelerated confidence intervals for the indirect effect was done implementing Preacher-Hayes' SPSS macro and using 20,000 bootstrap samples (Preacher and Hayes, 2008a). This analysis showed that empathic concern indeed mediated (95% CI [0.0001, 0.0075]) the relation between trait alexithymia and higher endorsement of utilitarian solution for personal moral dilemma (see Figure 1). Index of mediation (Preacher and Hayes, 2008b) for empathic concern, which is a standardized effect size measure of mediation, was 0.0148 (95% CI [0.0006, 0.0448]). Similar results emerged even after running the same mediation analysis controlling for age and gender (see Supplementary Figure S1). Thus, trait alexithymia influenced moral judgments via empathic concern such that it led to reduction in empathic concern which reduced the affective aversion to the prospect of personally harming someone for the greater good. Our third prediction was therefore also borne out by the data.

## Discussion

Recent dual process models of moral judgments posit automatic emotional intuitions that support deontological decisions which compete with response from deliberative reasoning systems that support utilitarian decisions on personal moral dilemmas. One source of negative affect that drives intuitive processes is outcome aversion arising from empathic response to the prospective distress that victim would be put through. There is overwhelming evidence to support the claim that reduction in this empathetic response can erode the negative affect making way for utilitarian judgments to come through and dominate the decision-making procedure (see Section Two Paths to Utilitarian Moral Judgments). Current results add to this rich body of literature another instance where empathic deficits lead to utilitarian moral judgments in alexithymic personalities.

### Alexithymia and empathy

Empathy entails the ability to *know* what others feel and to vicariously *experience* what others feel while keeping in mind that the target of this process is different from the self. Self-awareness is crucial to these abilities because one has to recognize one's own emotions to identify with those emotions in others (Hooker et al., 2008) or, to borrow words from Jonason and Krause (2013), one has to know where one's own shoes are before putting oneself in someone else's shoes. Thus, given problems associated with alexithymia in recognizing, identifying, labeling emotions (Nemiah et al., 1976) and their reduced ability to reflect on their internal states (Silani et al., 2008), it is unsurprising that they tend to have reduced empathic skills which have been extensively reported in the literature.

In the current study, we replicated results from previous studies (Guttman and Laporte, 2002; Moriguchi et al., 2007; Grynberg et al., 2010; Jonason and Krause, 2013) showing that trait alexithymia in general population is associated with (a) reduced empathic concern, (b) reduced perspective taking, (c) increased personal distress, but (d) shows no impairment on fantasy subscale of IRI. After ensuring that trait alexithymia indeed showed negative correlation with empathy, we explored how theses empathic deficits impacted decision-making in interpersonal domain by studying their moral judgments on moral dilemmas.

### Empathy and moral judgments

Since trait alexithymia is associated with reduced empathic skills, it provides a good testing ground to explore the exact link between empathy and morality. Although it has been argued that empathy is not necessary for moral judgments (Prinz, 2011) where there is no clearly discernible victim, e.g., insider trading, its epistemic and motivational role in harm-based moral judgments is undeniable (Pizarro, 2000; Ugazio et al., in press). Empathy has been argued to have led to evolution of harm/care-based morality (Decety, 2014) and is said to be essential for proper moral development (Hoffman, 2001), especially for developmentally learning the socio-moral norms about harm/care as opposed to other conventional norms (Blair, 1995). Empathy enables people to share the affective state of victims of others' moral actions. If this is an action that leads to distress in the victim, then empathic resonance with the victim's suffering leads to empathic arousal in the observer and informs her that morally relevant event is taking place (epistemic role) and motivates observer to either approach the victim to alleviate her suffering or to withdraw from the situation to remove the source of personal distress (motivational role). Thus, empathy is implicated as a moral emotion because it is a moral marker by which people understand that moral norms are being violated and are motivated to deem those actions morally wrong which cause suffering in the victim. This explains how people judge hypothetical situations where they are implicated as agents.

When people have to morally judge certain hypothetical action, they engage in evaluative simulation (Miller and Cushman, 2013) by putting themselves at the receiving end of the action (cognitive empathy) and experiencing what the patient would experience (affective empathy), which provides them with direct feedback about the consequences that such action would entail and people use this feedback of approbation or disapprobation as the motivational basis of their moral judgments (Ugazio et al., in press). For example, in case of footbridge dilemma, when participants simulate pushing the proximate large person to his/her death, they instantly experience an intense pang of negative emotion in response to suffering of the victim which prompts them to say no. Reduction in this empathy-based affective aversion to bad outcomes leads to more utilitarian moral judgments, while intensification of this aversion leads to more deontological moral judgments on personal moral dilemmas (see Section Two Paths to Utilitarian Moral Judgments). On the other hand, in impersonal moral dilemmas, the action of flipping switch, which changes the direction of toxic fumes and ultimately kills one person, is not as psychologically near (Liberman et al., 2007) and as personal (Greene et al., 2009) as pushing salient victim with one's own muscular force and, thus, does not elicit robust empathic response.

In this framework, it is easy to see how reduced empathic concern in trait alexithymia leads to deeming personally killing one to save many as more appropriate. When participants with higher score on trait alexithymia are presented with the option of pushing a stranger in footbridge dilemma, the reduced other-oriented feelings of compassion and warmth in response to the experience of this salient victim (as indexed by empathic concern) pre-empts the source of negative affect originating in affective aversion to distress in the victim (outcome aversion). Without this prepotent and robust affective reaction, the deliberative reasoning processes lead the charge and support the utilitarian solution. On the other hand, victim in the impersonal moral dilemma needs to be harmed in a psychologically distant way and hence the suffering and distress in this victim is construed at an abstract level as compared construal of victim suffering in the personal moral dilemma at a very concrete level (Eyal and Liberman, 2012). Since there is not strong empathic resonance with the psychologically distant victim (Liberman et al., 2007) and impersonal way of implementing harm is emotionally less salient (Greene et al., 2009), role of empathy-based outcome aversion is downplayed on impersonal moral dilemmas and reduced empathic concern in trait alexithymia does not lead to higher tendency to endorse utilitarian solution.

These results comport well with previous finding from study by Gleichgerrcht and Young (2013) that showed that intransigent utilitarians, i.e., people who endorsed utilitarian solution for both impersonal and personal dilemmas, scored low *only* on empathic concern and no other subscale of IRI than flexible utilitarians (who consented to utilitarian solution on impersonal but not personal dilemma) and nonutilitarians (who refused to accept utilitarian solution on any dilemma).

### Alternative explanations and future scope

Although we have argued here that reduced empathic concern leads to increased utilitarian judgments in trait alexithymia, we haven't ruled out the possibility that there might be some additional features of trait alexithymia that predisposes it toward a utilitarian moral profile. One can argue that in addition to reduced empathic concern, alexithymic personalities may also have higher preference for need for cognition and reflective thinking and this contributes to the utilitarian moral judgments (see Section Two Paths to Utilitarian Moral Judgments). Although we can't completely rule out this possibility, it seems unlikely given the evidence that trait alexithymia actually shows negative correlation with need for cognition (Bagby et al., 1986), which would predict *reduced* and not enhanced utilitarian tendencies in alexithymia.

Another plausible explanation is provided by Koven (2011) who showed that the construct clarity of emotion (i.e., how clearly one understands and can discriminate among one's emotions), was negatively correlated with utilitarian outcomes, while the construct attention to emotion (i.e., the degree to which one monitors and thinks about one's emotions) did not show any relation to utilitarian inclinations. She argues, based on research by Gohm (2003), that people who suffer from chronic confusion about their intense emotional experiences generally develop a trait-like pattern of down-regulating negative affect proficiently. Thus, in Koven's study, people with less clarity of emotion might have utilized negative affect elicited by personal moral dilemmas to lesser extent by down-regulating it and endorsed utilitarian judgments. Given that trait alexithymia is associated with confusion over one's emotional experience, it can be argued that they develop emotion regulation strategy of suppressing emotions which indeed seems to be the case (e.g., Swart et al., 2009). In other words, alexithymic people modulate their responses by expressive repression strategy in which emotion-expressive behavior is inhibited. Add to this the role of emotion regulation in moral judgments (Szekelya and Miu, 2014; Helion and Pizarro, in press) and it can be extrapolated that trait alexithymia harbors utilitarian proclivities due to additive effect of blunt negative affect owing to reduced empathic concern for the victim *and* suppression of this remaining affect due to superior cognitive control of emotion. Future brain imaging studies should be able to arbitrate on the validity of this line of reasoning.

### Limitations

One conspicuous shortcoming of this study is that sample consisted mostly of the young college-students, which limits the generalizability of these findings. It would be interesting to see if the same results can be replicated with clinical patients, e.g., people with psychosomatic complains, who present with high level of alexithymia.

Another shortcoming of the study is that we did not collect response time data and could not rule out participants who completed task insincerely based on this data. That said, we contend that it is unlikely that participants gave random responses to the questions because participants voluntarily logged onto the webpage to complete the survey and did not have any motivation apart from the wish to participate in the experiment.

Another drawback of the study is the limited number of moral dilemmas used, although this is not an unusual practice (e.g., Mendez et al., 2005; Gleichgerrcht et al., 2011, 2013; McIlwain et al., 2012; Trémolière et al., 2012; Gleichgerrcht and Young, 2013; Jack et al., 2014; Trémolière and Bonnefon, 2014).

Additional statistical worry is that the main results might be false positives. The sensitivity analysis (see Section Data analysis) showed that our study could detect correlation coefficient greater than 0.153 and yet we detected significant correlations (see Supplementary Tables S2,3) of −0.125 (TAS and empathic concern), 0.140 (TAS and ratings on personal moral dilemma), and −0.119 (empathic concern and ratings on personal moral dilemma). Although we can't completely rule out this possibility, we establish the stability of our results by computing confidence intervals for estimators of interest (correlation coefficients, regression coefficients, etc.) using bootstrapping methods with as many as 10,000 samples (minimum recommended: 1000; IBM SPSS Bootstrapping 20.0 manual, 2011). Bootstrapping methods are valuable when the sample sizes are small and are not representative of the entire population because they are asymptotically more accurate than the standard intervals computed based on sample variance and normality assumptions (Adèr et al., 2008). To sum it up, although we did not have sufficient sample size to detect significant associations that we did detect, we prop these results up with more reliable statistical techniques.

### Implications

Alexithymia has been shown to be comorbid with autism, with as many as 50% of autistic population exhibiting clinical scores on alexithymia measures (Hill et al., 2004). Additionally, autistic population has been shown to be more utilitarian than control population on personal moral dilemma (Gleichgerrcht et al., 2013), which was argued to be due to reduced cognitive empathy in autism. But, based on the current findings, alternative explanation for these results can be that autism is associated with utilitarian bias due reduced affective empathy owing to co-occurring alexithymia and will not be associated with any increased utilitarian tendency if individual alexithymia scores are entered as a covariate in the analysis (cf. Bird et al., 2010). Future studies should explore this possibility.

## Conclusion

This research provided additional evidence for a link between trait alexithymia and empathy deficits and explored how this disruption translated into behavior in hypothetical moral situations. Our findings suggest that this impairment in empathic skills, especially empathic concern, contributes to reduction in harm aversion which leads to increased propensity in alexithymic population toward agreeing to personally harming someone for the greater good on moral dilemmas. Results also provide additional evidence for the validity of Greene's dual process model for moral decision-making.

### Conflict of interest statement

The authors declare that the research was conducted in the absence of any commercial or financial relationships that could be construed as a potential conflict of interest.
