# Supplementary material for: Reduced empathic concern leads to utilitarian moral judgments in trait alexithymia
Source: Front Psychol. 2014 May 26;5:501. doi: 10.3389/fpsyg.2014.00501 (PMC4033264; doi:10.3389/fpsyg.2014.00501)
Supplement: Supplementary file 1 [file DataSheet1.DOCX]

**Supplementary material for “Reduced empathic concern leads to utilitarian moral judgments in alexithymia”**

Indrajeet Patil^*^, Giorgia Silani

Scuola Internazionale Superiore di Studi Avanzati, Neuroscience Sector, Trieste, Italy.

*Email: [ipatil@sissa.it](mailto:ipatil@sissa.it)

**Supplementary Text S1**

Detailed description of the two dilemmas used.

**Standard Fumes (impersonal moral dilemma)**

You work as the night caretaker in a small provincial hospital. During one of your rounds you realise that, because of a laboratory accident, some highly toxic fumes are spreading through the ventilation system towards a room in which there are five patients. In another room in the same ward there is just one patient.

You can activate a switch which allows the toxic fumes to be diverted away from the room with five patients. You know that the fumes will be directed to the single room where the patient will die, but the other five will be saved.

How appropriate is it for you to divert fumes to this room and sacrifice this one patient to save other five patients?

**Footbridge (personal moral dilemma)**

You are crossing a bridge which passes over the railway line and you see a fast-moving trolley on the track below. You notice that the trolley is out of control and that it will continue on its present course and collide with five people who are working ahead on the track, killing them. On the bridge next to you is a person you do not know and who happens to be very large.

You can throw this person off the bridge in such a way that this person’s large body will block the oncoming trolley on collision. You know that this person will die, but the five workers will be unhurt.

How appropriate is it for you to push this stranger to death in order to save the five workers?

**Supplementary Text S2**

In the main text, we provide results by treating alexithymia as a dimensional personality construct. Here, we will provide the same analysis by treating alexithymia as a categorical construct by dividing our sample into three groups: alexithymic (TAS-20 ≥ 61, *n* = 30), possibly alexithymic (52 ≤ TAS-20 ≤ 60, *n* = 60), and non-alexithymic (TAS-20 ≤ 51, *n* = 241). Since sample size for alexithymic and possibly alexithymic groups are too small as compared to non-alexithymic group, we combine alexithymic and possibly alexithymic into one group and compare variables of interest for this group with non-alexithymic group. We use Welch’s t-test for this analysis because it does not assume homogeneity of variance and corrects test statistic for unequal sample sizes (Welch, 1947).

*Note:* F = fantasy; PT = perspective taking; PD = personal distress; EC = empathic concern. 95% bias corrected and accelerated confidence intervals for correlation coefficient were generated using 10,000 bootstrap samples.

Thus, as in the main analysis, alexithymic/possibly alexithymic group reported to feel more personal distress and judged utilitarian moral judgment for personal moral dilemma to be more morally appropriate as compared to non-alexithymic group. Surprisingly, empathic concern did not differ between these two groups. But we contend that this is due to throwing away valuable variance by converting a continuous, dimensional variable (TAS-20) into a discrete, categorical one (alexithymic/possibly alexithymic, non-alexithymic). This dichotomization practice is rarely defensible and often yields misleading results and thus these results should not be trusted (MacCallum et al., 2002).

**Supplementary Table S1**

*Spearman rank correlations between TAS, IRI subscale scores and ratings on moral dilemmas with 95% confidence intervals for correlation coefficients.*

*Note:* TAS = Toronto Alexithymia Scale; F = fantasy; PT = perspective taking; PD = personal distress; EC = empathic concern. **p* < 0.05. ***p* < 0.01. ****p* < 0.001. 95% bias corrected and accelerated confidence intervals for correlation coefficient were generated using 10,000 bootstrap samples.

**Supplementary Table S2**

*Partial Spearman rank correlations controlling for age and gender (dummy coded 0: male, 1: female) between TAS, IRI subscale scores and ratings on moral dilemmas.*

*Note:* TAS = Toronto Alexithymia Scale; F = fantasy; PT = perspective taking; PD = personal distress; EC = empathic concern.

**p* < 0.05. ***p* < 0.01. ****p* < 0.001.

**Supplementary Table S3**

*Alexithymia (TAS) scores predicting ratings on moral dilemmas and empathy IRI subscales with additional predictor variables of age and gender.*

*Note*: TAS = Toronto Alexithymia Scale; F = fantasy; PT = perspective taking; PD = personal distress; EC = empathic concern; CI = confidence interval.

* 95% bias corrected and accelerated confidence intervals for logit coefficients were generated using 10,000 bootstrap samples. Positive or negative value of logit coefficient denote that increase in value of predictor variable is associated with increased odds for *higher* or *lower* value of criterion variable, respectively.

**Supplementary Table S4***IRI subscale scores predicting ratings on moral dilemmas in addition to age and gender.*

*Note*: F = fantasy; PT = perspective taking; PD = personal distress; EC = empathic concern; CI = confidence interval.

* 95% bias corrected and accelerated confidence intervals for logit coefficients were generated using 10,000 bootstrap samples. Positive or negative value of logit coefficient denote that increase in value of predictor variable is associated with increased odds for *higher* or *lower* value of criterion variable, respectively.

**Supplementary Figure S1**

Mediation analysis results. Negative logit coefficient from ordinal regression denotes reduced empathic concern and increased acceptability of utilitarian option on personal moral dilemma, while controlling for effects of age and gender. Bias-corrected and accelerated 95% CIs from 20,000 bootstrap samples are reported for specific indirect effects. Index of mediation (Preacher and Hayes, 2008) for this analysis is not provided because this option is currently unavailable for mediation analysis with covariates in Precher-Hayes’ macro.


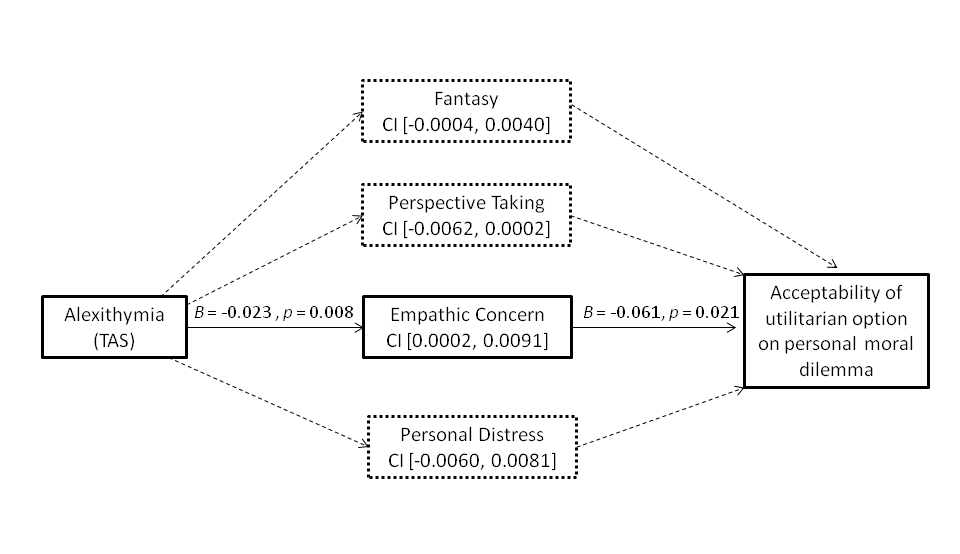


**Supplementary Figure S2**

Scatterplot for utilitarian moral judgments on personal moral dilemma (1: not at all morally appropriate, 7: very much morally appropriate) and alexithymia scores. Odds of higher endorsement of utilitarian solution increased from low to high alexithymia scores (see Table 2 of main text).


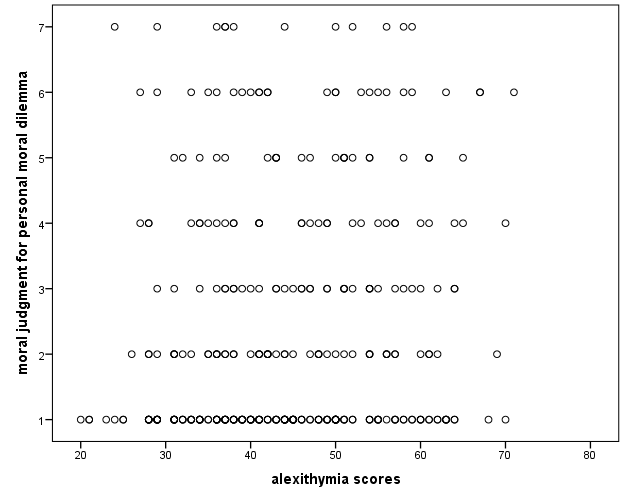


**References**

MacCallum, R. C., Zhang, S., Preacher, K. J., and Rucker, D. D. (2002). On the practice of dichotomization of quantitative variables. *Psychol. Methods*, 7, 19–40. doi: 10.1037/1082-989x.7.1.19

Preacher, K. J., and Hayes, A. F. (2008). Contemporary approaches to assessing mediation in communication research. In A. F. Hayes, M. D. Slater, and L. B. Snyder (Eds.), *The Sage sourcebook of advanced data analysis methods for communication research* (pp. 13–54). Thousand Oaks, CA: Sage.

Welch, B. L. (1947). The generalization of "Student's" problem when several different population variances are involved. *Biometrika*, 34 (1–2): 28–35. doi:10.1093/biomet/34.1-2.28
